# Mortality cost of sex-specific parasitism in wild bird populations

**DOI:** 10.1038/s41598-020-77410-6

**Published:** 2020-12-02

**Authors:** José O. Valdebenito, András Liker, Naerhulan Halimubieke, Jordi Figuerola, Tamás Székely

**Affiliations:** 1 Milner Centre for Evolution, Department of Biology and Biochemistry, University of Bath, Bath, UK; 2 MTA-PE Evolutionary Ecology Research Group, University of Pannonia, Veszprém, Hungary; 3 Behavioural Ecology Research Group, Center for Natural Sciences, University of Pannonia, Veszprém, Hungary; 4 Department of Wetland Ecology, Estación Biológica de Doñana (EBD-CSIC), Seville, Spain; 5 CIBER Epidemiología y Salud Pública (CIBERESP), Seville, Spain; 6 Department of Evolutionary Zoology and Human Biology, University of Debrecen, Debrecen, Hungary

**Keywords:** Sexual selection, Animal behaviour

## Abstract

Sex-specific mortality is frequent in animals although the causes of different male versus female mortalities remain poorly understood. Parasitism is ubiquitous in nature with widespread detrimental effects to hosts, making parasitism a likely cause of sex-specific mortalities. Using sex-specific blood and gastrointestinal parasite prevalence from 96 and 54 avian host species, respectively, we test the implications of parasites for annual mortality in wild bird populations using phylogenetic comparative methods. First, we show that parasite prevalence is not different between adult males and females, although Nematodes showed a statistically significant but small male-biased parasite prevalence. Second, we found no correlation between sex-biased host mortalities and sex-biased parasite prevalence. These results were consistent in both blood and gastrointestinal parasites. Taken together, our results show little evidence for sex-dependent parasite prevalence in adults in wild bird populations, and suggest that parasite prevalence is an unlikely predictor of sex difference in adult mortalities, not withstanding sampling limitations. We propose that to understand causes of sex-biased mortalities, more complex analyses are needed that incorporate various ecological and life history components of animals life that may include sex differences in exposure to predators, immune capacity and cost of reproduction.

## Introduction

Although sex ratio at birth is often close to 1:1 in wild populations, adult (or tertiary) sex ratios (the proportion of males to females in a population) are highly variable suggesting that sex differences in post-birth maturation, mortalities and/or population movements drive skewed adult sex ratios (ASR)^1–4^.

Mortality is a complex process, influenced by many factors that in simple terms could be classified as intrinsic and extrinsic to the individual (for example, immune capacity and ambient environment, respectively^5^). Predation, disease and starvation are important causes of mortality in wild animal populations, whereas body size and sexual selection are general predictors of mortality according to life history theory^6,7^, with larger animals often dying at lower rates than smaller ones^8–11^. Furthermore, social activities such as competition for food and/or mates may increase mortality of one sex more than the other^12–14^.

One important cause of mortality are pathogens or infectious agents. For instance, the history of the modern human has been marked by diseases of epidemic scale that resulted in millions of deaths that were caused by bacteria, viruses and parasites^15^. Most recently, the COVID-19 pandemic, although with relatively low mortality, showed to be more lethal for men than for women^16^. In wild animals, examples of elevated mortality due to pathogen infection often include native species exposed to exotic pathogens, driving populations to critically low numbers (e.g. Darwin’s finches^17^, Serengeti’s wild dogs and lions^18^) or even to the edge of extinction^19^. Moreover, pathogens have shown to also provoke mortality not by directly killing the host but debilitating and deteriorating their overall condition, increasing the chances of predation^20–23^.

Interestingly, despite the presumed relationship between pathogens (i.e. biological agent that causes disease or illness) and mortality in animals, information on the relationship between sex-biased infections and biased sex ratio is scarce. A notable exception occurred in mammals, where Moore and Wilson^1^ found a positive correlation across 106 mammal species for the bias in sexual size dimorphism (SSD) and the sex bias in parasitism, and that sex bias in parasitism predicted the sex bias in mortality, concluding that sexual selection for the larger sex (i.e. males) implicated a mortality cost through parasitism (see also^24^). Also, male mammals have a weaker immune competence, which correlates with higher presence of pathogens and mortality compared to females^25,26^. In birds, sex-biased infections and its implications on survival have not been assessed across a broad range of taxa, although indirect evidence would suggest so since a previous across-species meta-analysis found a male-biased prevalence of gastrointestinal parasites^27^. In addition, more recent complementary evidence shows that larger avian species are more affected by parasites, possibly because in larger hosts, parasites have greater space and niches to colonize and are likely to accumulate through life as larger species tend to live longer than smaller ones^9,28,29^. Therefore, from this perspective, and considering that in birds males are in general larger than females^30^, we could expect parasitism in birds to be male-biased. Although the difference in body size of birds is modest compared to mammals, it is unknown at what extent this could influence parasite load between the sexes. From a hormonal perspective, the sex hormones influence the reproductive behavior e.g. courting, territoriality, aggression, competition and nesting^31,32^, which could translate into sex-different rates of parasite acquisition due to immunosuppression mediated by testosterone or stress-released corticosterone (cost for males^25,33,34^), or sex-differences in behavior such as nesting time or breeding dispersal (cost for females^35,36^). However, current studies disregard the effect of sex hormones in immunity, as well as challenge the idea of sex-different immune response in birds^37,38^, finding little evidence supporting a significant effect of sex hormones in immunity in studies using physiological concentrations of hormones. Moreover, another study^26^ showed a lack of sex-differences in immunity across 241 immune estimates in birds, while recent evidence showed that, in general, immunosenescence also lacked sexual dimorphism across animals, including birds^39^.

Thus, the current evidence highlights males but not females as the sex more likely to be affected by parasites in birds^27,28,34^, although it is nevertheless unknown whether this variable could relate to the overall lower male mortality compared to females found in birds^13^, and suggested by their overall male-biased ASR^3,40^.

Nevertheless, studies using unsexed birds suggest an association between mortality and both blood and gastrointestinal parasitism, supported by evidence established through direct analysis of carcasses of mortality events or through capture-recapture survival analyses^41–44^. Moreover, blood (protozoan and microphilaria) and gastrointestinal parasites (helminths and coccidia) have different means of transmission that in turn could also influence patterns of sex-specific infection and thus mortality. For instance, nest type (open versus close) is often considered a risk factor for malaria infection because open-nesting offers increase exposure to dipteran vectors such as mosquitoes^45^.

To examine the relationship between sex-specific parasite prevalence and mortality, we obtained data from a total of 138 bird species (across 96 species from 13 avian orders for blood parasites and 54 species from 9 orders for gastrointestinal parasites) from published literature to test two hypotheses using phylogenetic comparative analyses. We use parasite prevalence because it gives an estimation of the infection status of a population, thus providing hints of their susceptibility to parasite infection (although not without limitations^46^). Also, determinants of parasite prevalence depend on a number of ecological and behavioral variables^47^ that could differ between the sexes^48^, as well as being one of the most commonly available parasite estimates in parasitology and ecology. First, we investigate whether males had higher parasite prevalence compared to females, as predicted by male’s modest but significantly larger body size^30^, male’s frequent stress-inducing behavior (corticosterone mediated immunosuppression^32,34^), and as previously shown in across-species studies in mammals^1^ and in birds (particularly gastrointestinal parasites^27^). Second, we evaluate whether sex-specific parasite prevalence predicted sex-specific adult annual mortality. Specifically, we (i) test the effect of parasite prevalence on mortality in males and females separately as they present variation in their physiology and life histories^49^ that could influence the degree of exposure and/or infection to parasites and subsequent mortality^48,50^. Finally, we (ii) evaluate whether sex-specific adult annual mortality is predicted by sex-specific adult parasite prevalence, including SSD and mating competition in the analysis as potential confounding variables^24,51,52^.

## Material and methods

### Literature search

We collected data on sex-specific prevalence of parasitism in birds using ISI Web of Science and Google Scholar. The use of Google Scholar in systematic reviews has been recently criticized^53^, however, in our study we used Google Scholar because it expands searches to include grey literature, such as technical reports and theses. The searches were conducted by using the following keyword combinations: “scientific name of host species” + parasit*, prevale*, helmint*, blood, malar*, haemoparasit*, mite* or lice. Because our aim was to evaluate the effect of parasitism on sex-specific mortality, the list of names searched initially corresponded to 369 bird species included in the dataset of sex-specific annual mortality data provided by Székely et al.^52^. If the bird species name had synonyms, the search was repeated with every name. The references of previous reviews and meta-analyses were also checked (see supplementary material). The inclusion criteria required the parasite prevalence to be: (i) determined from adult birds with known sex, (ii) obtained from wild birds (not captive), and (iii) from infection naturally acquired (not experimentally infected). We only included studies reporting results for both males and females to avoid difficulties comparing prevalences within species generated by different sampling/diagnostic methods or different populations. We included studies with haemoparasite detection through molecular and optic microscopy methods because both bring comparable results and to date there is not consensus about which technique is better over the other^54,55^. All studies available for gastrointestinal and external parasites used exclusively taxonomic keys diagnosis through microscopic examination. Studies based on parasite’s egg counts were not considered to minimize the chances of including studies containing false negative results originated by the variation in egg shedding rhythms seen in some gastrointestinal parasites^56^. In order to obtain a robust estimate of parasite prevalence for a given host species, all publications that met the inclusion criteria were included in our dataset. Further details of the literature search as well as the full list of studies consulted are given in the supplementary material (Tables S1 and S2).

### Body mass, adult mortality and sexual competition

Data on sex-specific body mass, annual adult mortality and sexual competition were obtained from Székely et al.^52^. Data were augmented following the method provided by Székely et al.^52^ and Liker et al.^57^, consisting of searching the name of the additional bird species in scientific citation indexes, books, species monographs and electronic databases (see supplementary material). We included mortality estimates obtained from field studies in which the estimates for both males and females were determined in the same population and with the same method. Three main methods were used to determine mortality rates: capture-recapture, ringing recoveries and local return rates. Mating system was determined as a five-point score by the frequency of polygamy for each sex, with “0” corresponding to very rare or no polygamy, “1” to rare polygamy, “2” to uncommon polygamy, “3” to moderate polygamy and “4” to common polygamy (for more details see^57^).

### Parasite prevalence

The final dataset included 96 bird species (sample size range of 4–1045) with sex-specific blood parasite prevalence data, 54 species (5–9729) with gastrointestinal parasite prevalence data and only 3 species (13–131) with ectoparasite prevalence data. Ectoparasites were excluded from further analyses due to the low sample size. Blood parasites were divided into five categories: *Haemoproteus*, *Leucocytozoon*, *Plasmodium*, *Trypanosoma* and Microfilaria. Whereas gastrointestinal parasites were categorized as Cestoda, Acanthocephala, Nematoda, Trematoda and Protozoa. Finally, one last category received data presented as the combination of two or more parasite categories (for example, we often found blood parasite studies reporting the overall prevalence of *Haemoproteus*, *Leucocytozoon* and *Plasmodium*, three parasites categories combined in one single datum). Some studies of blood parasitism included avian species that presented 0% prevalence in both sexes. These studies were included in the dataset, although it was uncertain whether birds never got parasitized due to vector absence in their habitats^58^, were able to reduce parasitemia under detection limits, or because the parasites were unable to complete their life-cycle in the host^59^. Along with parasite prevalence data we also recorded the period of the year when parasites were samples, which was divided in three categories: breeding (sampling took place mostly during the hosts' breeding period), nonbreeding (sampling took place outside the breeding period) and year-round (sampling included both breeding and nonbreeding periods).

### Phylogenetic meta-analysis

To investigate sex difference in parasite prevalence, a phylogenetic multilevel meta-analysis was performed using the R package *metafor*^60^. Because all studies only provided prevalence and sample size values, we opted to group the birds as infected and not infected males and females in 2 × 2 contingency tables and then calculate the effect size as log odds ratio^60^. We conducted the meta-analyses including period of sample (breeding, nonbreeding and year-round) and method of parasite detection (only for blood parasites, consisting of three categories: molecular detection, optic microscopy detection, and both) as moderators (i.e. fixed-effect), and study and phylogeny (a variance–covariance matrix) as random-effect variables.

Publication bias (due to missing studies that were not published because of negative or null results^61^) was evaluated using Egger’s regression test^62,63^ by including the standard error of the effect sizes as an additional moderator within the model. If the intercept significantly deviated from zero (significance of *P* < 0.10^62^) the overall relationship between the precision and size of studies included in the dataset was considered asymmetrical, or in other words, biased^63^. Of the twelve models conducted, two suggested presence of publication bias, corresponding to the gastrointestinal parasites Nematoda (*P* = 0.035) and Trematoda (*P* = 0.043). Diagnostic tests for identifying influential data points and outliers, and rules for excluding these types of cases are still evolving, particularly for multivariate/multilevel meta-analytical models^64^. To address this, our approach consisted of identifying the influential outliers causing the bias and running the models after excluding these values [see^65^].

Statistical power in random-effects meta-analysis can be difficult to determine. It has been suggested that, in general, meta-analyses with at least five studies offer more power than the individual studies alone^66^. Therefore, outcomes below this five-studies threshold should be taken carefully.

### Phylogenetic comparative analysis

We used phylogenetic generalized least squares (PGLS) to test whether parasite prevalence was related to annual mortality, adult body mass and sexual competition. This approach allows controlling for the non-independence among species by incorporating a variance–covariance matrix that represents their phylogenetic relatedness^67^. In all models we used Pagel’s lambda (λ) as measure of phylogenetic signal^68^ and it was set to the maximum-likelihood value^69^. Prior to the analyses, prevalence and mortality were logit-transformed^70^. Mortality bias was expressed as log(male mortality/female mortality). Average body mass (in grams) of male and female adults was log-transformed, whereas SSD in adult body mass was expressed as log(male body mass (g)/female body mass (g)). The sex bias in mating system was calculated as the difference between male and female polygamy scores^57^. Because often each host species had several estimates of prevalence (i.e. studies reporting estimates for more than one parasite group), the sex bias in parasite prevalence of each bird species was incorporated into this analysis as the weighted average effect size of all comparisons. Instances where multiple studies reported prevalence estimates for the same host species were handled by adding sister tip labels (of the same branch length) to the phylogeny. The effect size per species was calculated using the function *escalc* of the R package *metafor* with log odds ratio as measure. We fitted both single-predictor and multi-predictor models to blood parasites and gastrointestinal parasites and each model was run separately for females, males and sex bias. To account for phylogeny, we used the avian phylogeny from Jetz et al.^71^. The analyses were run using consensus trees (one for each type of parasitism, Fig. S1) obtained through the method 50% majority-rule^72,73^ from 1,000 randomly selected trees from a pool of 10,000 available (https://birdtree.org), using the methodology described by Rubolini et al.^74^. These phylogenetic trees were not fully resolved, and polytomies were arbitrarily resolved by adding a branch distance of 10^–08^ to one randomly chosen branch in the polytomy using the function *multi2di* from the R package *ape*^75^. All PGLS analyses were conducted in R using the package *caper*^76^.

## Results

### Phylogenetic meta-analysis

**Figure 1:**
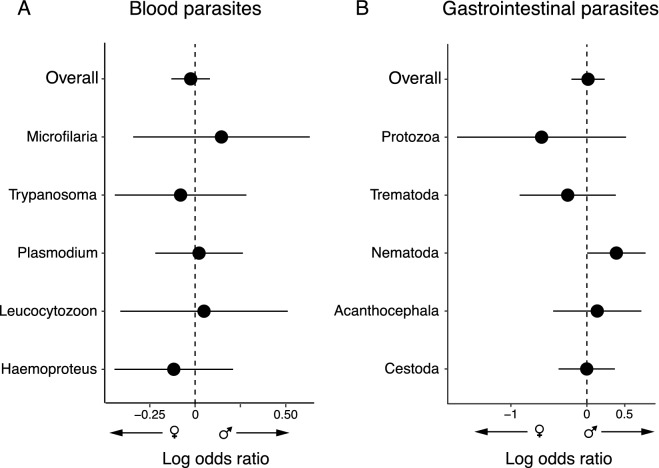
Sex bias in prevalence of (**A**) blood parasites and (**B**) gastrointestinal parasites in birds. Weighted average effect size estimates, showing lower and upper 95% confidence intervals in overall meta-analyses and broken down results according to parasite category (see Material and methods). The dashed vertical line indicates no sex difference, positive values represent male bias prevalence and negative values female bias. See Table 1 for statistics.

Overall, males and females did not exhibit different prevalence of blood parasites nor gastrointestinal parasites (Fig. 1 and Table 1). In the analysis broken down for parasite category (five categories of blood parasites and five of gastrointestinal parasites; Table 1), only Nematodes showed a weak male-biased prevalence (Fig. 1b; *k* = 33, estimate = 0.388, *Z* statistic = 1.979, *P* = 0.048, 95% CI = 0.004, 0.773).

**Table 1:** Phylogenetic meta-analysis of sex difference in prevalence of blood parasites and gastrointestinal parasites. The estimate represents the weighted average effect size as log odds ratio and its positive or negative value represents the sex bias directionality (see Fig. 1).

	*Q*_REML_ (*P*-value)	*k*	*n*	Studies	Estimate (95% CI)	*Z* statistic (*P*-value)
**Prevalence of blood parasite (overall)**	265.994 (0.043)	229	96	78	− 0.024 (− 0.130, 0.082)	− 0.451 (0.652)
Haemoproteus	61.240 (0.575)	69	60	51	− 0.117 (− 0.444, 0.210)	− 0.704 (0.481)
Leucocytozoon	39.710 (0.6559)	49	43	33	0.049 (− 0.413, 0.511)	0.209 (0.835)
Plasmodium	30.820 (0.822)	44	39	29	0.022 (− 0.220, 0.263)	0.178 (0.859)
Trypanosoma	17.257 (0.8375)	28	23	21	− 0.080 (− 0.443, 0.283)	0.186 (0.666)
Microfilaria	5.186 (0.878)	13	10	10	0.145 (− 0.341, 0.632)	0.591 (0.555)
**Prevalence of gastrointestinal parasites** **(overall)**	226.818 (< 0.001)	116	49	37	0.016 (− 0.203, 0.234)	0.140 (0.889)
Cestoda	68.354 (< 0.001)	27	23	22	− 0.002 (− 0.372, 0.368)	− 0.011 (0.991)
Acanthocephala	6.141 (0.726)	12	10	10	0.137 (− 0.444, 0.717)	0.461 (0.645)
Nematoda	37.544 (0.162)	33	22	20	0.388 (0.004, 0.773)	3.918 (0.048)
Trematoda	20.086 (0.389)	21	11	8	− 0.252 (− 0.885, 0.380)	− 0.782 (0.434)
Protozoa	12.537 (0.484)	15	15	5	− 0.596 (− 1.708, 0.516)	− 1.050 (0.294)

Meta-analyses were performed using multilevel random-effect meta-analysis with restricted maximum likelihood (REML). Fixed-effect variables: period of sampling and method of parasite detection. Random-effect variables: phylogenetic relatedness and study. *Q*_REML_ = test for heterogeneity; *k* = number of effect sizes; *n* = number of host species; Studies = number of studies.

### Parasite prevalence and annual adult mortality

**Figure 2:**
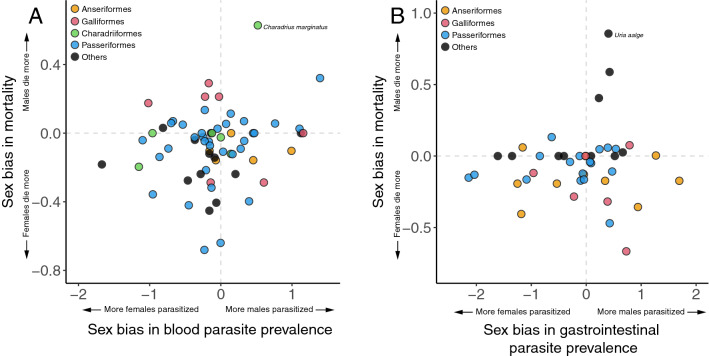
Sex bias in annual mortality in relation to the sex bias in prevalence of (**A**) blood and (**B**) gastrointestinal parasites (see Table 2 for statistics). Sex bias in mortality was expressed as log(male mortality/female mortality), whereas the sex bias in parasite prevalence was expressed as the weighted average effect size of all comparisons (see Material and methods). Represented in colors are the avian orders with the greatest numbers of species in each of the analyses (full species list in supplementary material). Outliers are specified. Dashed lines indicate no sex difference, positive values represent male bias and negative values female bias.

We found no association between annual mortality and prevalence in either blood parasites or gastrointestinal parasites (Table 2). The lack of association was consistent when each sex was tested separately (Table 2) and also when analyzing the sex bias (Fig. 2 and Table 2).

**Table 2:** Phylogenetic generalized least squares (PGLS) showing single-predictor and multi-predictor relationships between annual mortality and prevalence of (a) blood parasites and (b) gastrointestinal parasites. Multi-predictor models include two additional life history variables: body mass and mating system. First each sex was analyzed separately, then we tested the relationship between sex bias in the response and predictor variables (see Material and methods).

Response variable	Explanatory variable	Slope	*P*
(a) Blood parasites			
*Single-predictor models (n = 63)*			
Male annual mortality Adjusted *R*^2^ = 0.00; λ = 0.805	Male overall blood parasite prevalence	0.009	0.899
Female annual mortality Adjusted *R*^2^ = 0.00; λ = 0.735	Female overall blood parasite prevalence	0.039	0.560
Sex bias in annual mortality Adjusted *R*^2^ = 0.02; λ = < 0.001	Sex bias in blood parasite prevalence	0.064	0.167
*Multi-predictor models*			
Male annual mortality (n = 56) Adjusted *R*^2^ = 0.18; λ = 0.994	Male overall blood parasite prevalence	0.021	0.554
	Male body mass	− 0.208	0.002
	Male mating system	0.109	0.025
Female annual mortality (n = 55) Adjusted *R*^2^ = 0.18; λ = 0.925	Female overall blood parasite prevalence	0.018	0.718
	Female body mass	− 0.242	0.007
	Female mating system	0.252	0.045
Sex bias in annual mortality (n = 55) Adjusted *R*^2^ = 0.03; λ = < 0.001	Sex bias in blood parasite prevalence	0.046	0.223
	Sexual size dimorphism	0.154	0.159
	Sex bias in mating system	0.029	0.056
(b) Gastrointestinal parasites			
*Single-predictor models (n = 43)*			
Male annual mortality Adjusted *R*^2^ = 0.00; λ = 0.917	Male overall gastrointestinal parasite prevalence	− 0.008	0.889
Female annual mortality Adjusted *R*^2^ = 0.02; λ = 0.999	Female overall gastrointestinal parasite prevalence	0.055	0.173
Sex bias in annual mortality Adjusted *R*^2^ = 0.00; λ = 0.384	Sex bias in gastrointestinal parasite prevalence	0.034	0.414
*Multi-predictor models (n = 43)*			
Male annual mortality Adjusted *R*^2^ = 0.31; λ = 0.900	Male overall gastrointestinal parasite prevalence	0.013	0.791
	Male body mass	− 0.415	< 0.001
	Male mating system	0.138	0.130
Female annual mortality Adjusted *R*^2^ = 0.170; λ = 0.950	Female overall gastrointestinal parasite prevalence	0.005	0.913
	Female body mass	− 0.353	0.005
	Female mating system	0.107	0.435
Sex bias in annual mortality Adjusted *R*^2^ = 0.44; λ = 0.999	Sex bias in gastrointestinal parasite prevalence	0.007	0.749
	Sexual size dimorphism	− 0.936	< 0.001
	Sex bias in social mating system	− 0.009	0.779

These results remained qualitatively unchanged after conducting multi-predictor analyses incorporating body mass and mating competition into the models (Table 2). In these latter analyses only body mass and mating competition had a significant effect on mortality, although the relationship with mating competition was significant only in the blood parasite analyses (Table 2a).

In most cases the phylogenetic signal (**λ**) was moderate to high, indicating important variation associated to phylogenetic relatedness, however, further examination considering avian orders show no clear clustering for sex bias analyses (Fig. 2 and S2).

## Discussion

To our knowledge, this work represents the largest comparative study of sex-specific parasite prevalence in birds, based on 96 species with sex-specific blood parasite prevalence data and 54 species with gastrointestinal parasite prevalence data. Taken together, our results showed little evidence supporting sex biases in parasite prevalence, with no overall sex bias in blood or gastrointestinal parasites prevalence in birds. Additionally, no relationship was found between sex bias in mortality and sex bias in parasite prevalence, even after controlling for possible confounding life history variables, i.e. mating system, body size and sexual size dimorphism.

Our findings do not support the prediction of male-biased parasitism generated by the sexual size dimorphism^1,30^ and sex-different hormonal immunosuppression^31,33^. One possible explanation is that in birds the magnitude of the difference in size between sexes tends to be smaller compared to mammals^77^, where an association between sexual size dimorphism and parasite prevalence has been shown^1^. Furthermore, some evidence shows little and no relationship between body size and blood parasites across avian species^47,78,79^. Scheuerlein and Ricklefs^80^ found an association in parasite prevalence and body size in passerines, however, after controlling for phylogeny, the association was marginal. On the other hand, although stress and sex hormones were not part of our analysis, our results give little support to the idea of sex-differences in corticosterone immunosuppression, and seem to be in line of with recent research finding inconclusive results in the immunocompetence handicap theory in birds^26,37,81^.

Specifically, we found no sex bias in the overall prevalence of blood parasites, consistent with the overall results of a previous meta-analysis of blood parasites in birds^79^. Sex differences in blood parasites are generally thought to occur due to unequal exposure of the sexes to vectors^82,83^ and differences between males and females in the immune-endocrine system^84^. Perhaps the lack of sex differences seen here could be attributed to these processes balancing each other out. For example, in males, the persistent pressure of male-male competition could generate stress-induced corticosterone which due to its immunodepressive effect could make them more prone to infection^34^, at the same time that the elevated exposure of females to vectors while incubating^35^. Poulin^27^ found a strong male-biased infection of Acanthocephalan and Nematodes parasites, consistent with our results in the overall parasite prevalence in Nematoda. Nematodes are a very diverse group of round worms. Male-biased parasite prevalence in this group could be due to many non-exclusive variables including those previously suggested for overall gastrointestinal parasites (mainly based on differences in body size; see Introduction), in addition to sex-specific foraging behavior as result of niche specialization or competitive exclusion by the dominant sex^85,86^. However, more studies are needed to test these hypotheses.

Mortality was not related to parasite prevalence across all analyses conducted, even in multi-predictor analyses where mortality was tested against parasite prevalence, body mass and mating system. Only body mass was consistently associated with mortality as found in previous studies^13,52,87^. Although parasite burden has often been linked to mortality in species-specific studies in birds^23,44,88^ (but see^89^), here we found that such association seems to be less clear at interspecific level. Nevertheless, our results should be treated cautiously because in most cases parasitism and mortality data did not come from the same population, and because parasite data for males and females is more likely to be reported in studies investigating sexually dimorphic birds, therefore, we cannot discard a possible bias toward sexually dimorphic species over monomorphic ones. In addition, prevalence, as an index of parasitism, could be problematic because it informs about the proportion of infected individuals in relation to the number examined^90^, generating uncertainty whether the individuals found positive only correspond to infection-resistant animals that survived the infection^82^. For example, a previous study found that males had lower survival than females to influenza A virus infection^91^, therefore, in the hypothetical situation of sampling this population in the wild without knowing this sex-different viral susceptibility beforehand, and assuming a similar infection rate between sexes, females would have a higher prevalence than males because a larger proportion of infected males died.

In contrast to the findings of Moore and Wilson^1^ in mammals, sex-biased parasitism in birds did not seem to be a consistent driver of sex-specific mortality. The pressure that parasites impose on birds not only appeared to be low between sexes but also within sexes as no increase nor diminution of mortality were seen when tested males and females separately. Perhaps, juveniles should be the target by further studies to obtain a thorough understanding of mortality patterns. Accordingly, a recent study suggests that juvenile mortality rather than chick and adult mortality corresponded to the main contributor of sex biases in ASR in six plover populations (*Charadrius*)^92^. Unfortunately, juvenile sex-specific parasitism data in birds is scant.

In conclusion, our analyses showed that birds do not exhibit overall sexual difference in parasite prevalence, and parasite prevalence do not predict sex-specific mortality, thus suggesting that other processes may drive the sex-differences in adult mortalities reported from numerous bird species. Though, perhaps the limitations in our analysis (mentioned above) contributed to this lack of association. Although life history traits (e.g. mating system, parental care, and body mass) have been shown as important predictors of mortality in birds^13,52,87^, the actual etiology that originates female-biased mortality in birds is still poorly explored. Perhaps mortality events during migration^93^, predation^94^, susceptibility to stress^95^, or simply resilience to starvation are more important determining sex-specific mortality than parasites. In addition to this, understanding male versus female immune systems undoubtedly is highly relevant. We call for further comparative and single-species studies to understand the causes of sex different mortality patterns.

## Supplementary information

Supplementary information.

## Data Availability

The full list of references consulted to extract the parasite data is given in the supplementary material. The dataset and R code can be accessed on 10.6084/m9.figshare.13232435.v1.
