# Supplementary material for: Mortality cost of sex-specific parasitism in wild bird populations
Source: Sci Rep. 2020 Dec 2;10:20983. doi: 10.1038/s41598-020-77410-6 (PMC7710712; doi:10.1038/s41598-020-77410-6)
Supplement: Supplementary file 1 — Supplementary information. [file 41598_2020_77410_MOESM1_ESM.docx]

**Mortality cost of sex-specific parasitism in wild bird populations**

José O. Valdebenito^1^, András Liker^2,3^, Naerhulan Halimubieke^1^, Jordi Figuerola^4,5^ and Tamás Székely^1,6*^

^1^Milner Centre for Evolution, Department of Biology and Biochemistry, University of Bath, Bath, United Kingdom

2MTA-PE Evolutionary Ecology Research Group, University of Pannonia, Veszprém, Hungary

3Department of Limnology, University of Pannonia, Veszprém, Hungary

4Department of Wetland Ecology, Estación Biológica de Doñana (EBD-CSIC), Seville, Spain

^5^CIBER Epidemiología y Salud Pública (CIBERESP), Seville, Spain

6Departmen of Evolutionary Zoology and Human Biology, University of Debrecen, Debrecen, Hungary

*Corresponding author: Tamás Székely, e-mail: T.Szekely@bath.ac.uk

**Supplementary material**

A

B

**Figure S1.** Phylogenetic hypothesis used in the comparative analysis for (A) blood parasites and (B) gastrointestinal parasites.

A

B

**Figure S2.** Phylomorphospace plots of the association between the sex bias in annual mortality and (A) the sex bias in prevalence of blood parasites, and (B) the sex bias in gastrointestinal parasites. Represented in colors are the four and three avian orders with the greatest numbers of species in each of the analysis.

**Literature search**

Because our aim was to evaluate the effect of parasitism on sex-specific mortality, the list of names searched initially corresponded to 369 bird species included in the dataset of sex-specific annual mortality data provided by^47^ Székely et al. (Proc. R. Soc. B 2014; 281:20140342).

We found 78 studies that met the inclusion criteria for blood parasites and 39 for gastrointestinal parasites. These studies were result of searches on ISI Web of Science, Google Scholar, and consulting the material used in Poulin (Am. Nat. 1996; 147(2):287-295), McCurdy et al. (Oikos 1998; 82(2):303-312). Two studies corresponded to Master of Science theses and one to a Doctoral thesis. Details of the species and each corresponding study used in the analysis are given in Table S1 and S2.

After the initial search, we found blood parasite data for 96 bird species, 38 of which were not listed in Székely et al. (Proc. R. Soc. B 2014; 281:20140342). For these 38 species we collected mortality, mating system and body size data using the methodologies described in Székely et al. (Proc. R. Soc. B 2014; 281:20140342) and Liker et al. (Curr. Biol. 2014; 24(8):880-884). More details are given in the methods section, main text.

For gastrointestinal parasites, from the 54 species found, 7 were not included in Székely et al. (Proc. R. Soc. B 2014; 281:20140342). For these 7 species, data on mortality, mating system and body size was collected as described above.

**Table S1**. List of 78 studies consulted to extract data on blood parasites.

| species | order | study |
| --- | --- | --- |
| Falco sparverius | Falconiformes | Apanius, V. & Kirkpatrick, C.E. 1988. Preliminary report of Haemoproteus tinnunculi infection in a breeding population of American Kestrels. J. Wildl. Dis. 24(1):150-153. |
| Accipiter nisus | Accipitriformes | Ashford, R.W. et al 1990. Leucocytozoon toddi in British sparrowhawks Accipiter nisus: observations on the dynamics of infection. J. Nat. Hist. 23(5): 1101-1107. |
| Cardinalis phoeniceus | Passeriformes | Belo, N.O. et al. 2012. Diversity of avian haemosporidians in arid zones of northern Venezuela. Parasitology 139: 1021-1028. |
| Tiaris bicolor | Passeriformes | Belo, N.O. et al. 2012. Diversity of avian haemosporidians in arid zones of northern Venezuela. Parasitology 139: 1021-1028. |
| Grus americana | Gruiformes | Bertram, M.R. et al. 2016. Haemosporida prevalence and diversity are similar in endangered wild whooping cranes (Grus americana) and sympatric sandhill cranes (Grus canadensis). Parasitology 144(5): 629-640. |
| Grus canadensis | Gruiformes | Bertram, M.R. et al. 2016. Haemosporida prevalence and diversity are similar in endangered wild whooping cranes (Grus americana) and sympatric sandhill cranes (Grus canadensis). Parasitology 144(5): 629-640. |
| Zonotrichia leucophrys | Passeriformes | Bonier, F. et al. 2006. Sex-specific consequences of life in the city. Behav. Ecol. 18(1): 121-129. |
| Anthus campestris | Passeriformes | Calero-Riestra, M. & García, J.T. 2016. Sex-dependent differences in avian malaria prevalence and consequences of infections on nestling growth and adult condition in the Tawny pipit, Anthus campestris. Malaria Journal 15: 178. |
| Pica nuttalli | Passeriformes | Clark, G. 1964. Frequency of Infection and Seasonal Variation of Leucocytozoon berestnefi in the Yellow-billed Magpie, Pica nuttalli. The Journal of Protozoology 11(4): 481-484. |
| Larus scopulinus | Charadriiformes | Cloutier, A. et al. 2011. Plasmodium infections of red-billed gulls (Larus scopulinus) show associations with host condition but not reproductive performance. J. R. Soc. N. Z. 41(4): 261 277. |
| Meleagris gallopavo | Galliformes | Cook, R.S. et al. 1966. Haemoproteus in Wild Turkeys from the Coastal Bend of South Texas. The Journal of Protozoology 13(4): 588-590. |
| Ficedula hypoleuca 2 | Passeriformes | Dale, S. et al. 1996. Effects of blood parasites on sexual and natural selection in the pied flycatcher. J. Zool. 238(2): 373-393. |
| Progne subis | Passeriformes | Davidar, P. & Morton, E.S. 1993. Living with parasites: prevalence of a blood parasite and its effect on survivorship in the purple martin. The Auk 110(1):109-116. |
| Junco hyemalis | Passeriformes | Deviche, P. et al. 2001. Seasonal and Age-Related Changes in BloodParasite Prevalence in Dark-Eyed Juncos (Junco hyemalis, Aves, Passeriformes). J. Exp. Zool. 289(7): 456-466. |
| Loxia leucoptera | Passeriformes | Deviche, P. et al. 2009. Blood parasitaemia in a high latitude flexible breeder, the white-winged crossbill, Loxia leucoptera: contribution of seasonal relapse versus new inoculations. Parasitology 137(2): 261-273. |
| Cygnus olor | Anseriformes | Dolka, B. et al. 2014. Hematological parameters in relation to age, sex and biochemical values for mute swans (Cygnus olor). Veterinary Research Communications 38: 93–100. |
| Ficedula hypoleuca | Passeriformes | Dubiec, A. et al. 2017. Haemoparasites of the pied flycatcher, inter-population variation in the prevalence and community composition. Parasitology 145(7): 912-919. |
| Parus major | Passeriformes | Dunn, J. et al. 2011. Personality and parasites: sex-dependent associations between avian malaria infection and multiple behavioural traits. Behav. Ecol. Sociobiol. 65:1459-1471. |
| Quelea quelea | Passeriformes | Durrant, K.L. et al. 2007 Variation in haematozoan parasitism at local and landscapelevels in the red-billed quelea Quelea quelea. J. Avian Biol. 38(6): 662-671. |
| Baeolophus bicolor | Passeriformes | Fast, K.M. et al. 2016. Haemosporidian prevalence and parasitemia in the tufted titmouse (Baeolophus bicolor). J. Parasitol. 102(6): 636-642. |
| Acrocephalus schoenobaenus | Passeriformes | Fernandez et al 2010 High prevalence of haemosporidians in Reed Warbler Acrocephalus scirpaceus and Sedge Warbler Acrocephalus schoenobaenus in Spain |
| Acrocephalus scirpaceus | Passeriformes | Fernandez, M. et al. 2010. High prevalence of haemosporidians in Reed Warbler Acrocephalus scirpaceus and Sedge Warbler Acrocephalus schoenobaenus in Spain. J. Ornothol. 151: 27. |
| Dendragapus obscurus | Galliformes | Forbes, M. R. et al. 1994. Blood Parasites of Blue Grouse: Variation in Prevalence and Patterns of Interspecific Association. Oecologia 97(4): 520-525. |
| Dendragapus obscurus 2 | Galliformes | Forbes, M. R. et al. 1994. Blood Parasites of Blue Grouse: Variation in Prevalence and Patterns of Interspecific Association. Oecologia 97(4): 520-525. |
| Passerculus sandwichensis | Passeriformes | Freeman-Gallant, C.R. et al. 2001. Sexual Selection and the Geography of Plasmodium Infection in Savannah Sparrows (Passerculus sandwichensis). Oecologia 127: 517-521. |
| Cyanocitta cristata | Passeriformes | Garvin, M. & Schoech, S. 2006. Hormone Levels and Infection of Haemoproteus danilewskyi in Free-Ranging Blue Jays (Cyanocitta cristata) J. Parasitol., 92(3): 659-662. |
| Anas discors | Anseriformes | Garvon, J.M. et al. 2016. Blood Parasites of Blue-winged Teal (Anas discors) from Two Migratory Corridors, in the Southern USA. 52(3): 725-729. |
| Zenaida macroura | Columbiformes | Godfrey R.D. et al. 1990. Effects of host and spatial factors on a haemoproteid community in mourning doves from western. J. Wildl. Dis. 26(4): 435-441. |
| Prunella collaris | Passeriformes | Haas, M. & Kisková, J. 2010. Absence of blood parasites in the Alpine Accentor Prunella collaris. Oecologia Montana 19: 30-34. |
| Arenaria interpres | Charadriiformes | Hargreaves, A.L. et al. 2010. Concentrations of 17 elements, including mercury, and their relationship to fitness measures in arctic shorebirds and their eggs. Science of the Total Environment 408(16): 3153-3161. |
| Carpodacus mexicanus | Passeriformes | Hartup, B.K. et al. 2008. Blood Parasites of House Finches (Carpodacus mexicanus) from Georgia and New York. J. Wildl. Dis. 44(2): 469-474. |
| Acrocephalus arundinaceus | Passeriformes | Hasselquist, D. et al. 2007. Temporal patterns of occurrence and transmission of the blood parasite Haemoproteus payevskyi in the great reed warbler Acrocephalus arundinaceus. J. Ornithol. 148: 401-409. |
| Turdus merula | Passeriformes | Hatchwell, B.J. et al. 2000. The prevalence and ecology of the haematozoan parasites of European blackbirds, Turdus merula. Canadian Journal of Zoology 78(4): 684-687. |
| Lagopus lagopus | Galliformes | Holmastad, P.R. & Skorping A. 1998. Covariation of parasite intensities in willow ptarmigan, Lagopus lagopus L. Canadian Journal of Zoology 76(8): 1581-1588. |
| Accipiter soloensis | Accipitriformes | Hsu, Y.C. et al. 2015. Prevalence of Blood Parasites in Three Migratory Raptor Species from Taiwan J. Raptor Res. 49(2): 227-230. |
| Butastur indicus | Accipitriformes | Hsu, Y.C. et al. 2015. Prevalence of Blood Parasites in Three Migratory Raptor Species from Taiwan J. Raptor Res. 49(2): 227-230. |
| Falco tinnunculus | Falconiformes | Hsu, Y.C. et al. 2015. Prevalence of Blood Parasites in Three Migratory Raptor Species from Taiwan J. Raptor Res. 49(2): 227-230. |
| Dendragapus canadensis | Galliformes | Jones, T.L. & Robinson, W.L. 1969. Blood parasites of Michigan spruce grouse, Canachites canadensis. J. Parasitol. 55(3): 492. |
| Falco tinnunculus 2 | Falconiformes | Korpimaki, E. et al. 1995. Blood parasites, sexual selection and reproductive success of European kestrels. Ecoscience 2(4): 335-343/ |
| Aegolius funereus | Strigiformes | Korpimdki, E. et al. 1993. Blood parasites and reproductive success of Tengmalm's owls: detrimental effects on females but not on males? Functional Ecology 7: 420-426. |
| Parus cinctus | Passeriformes | Krams, I. et al. 2010. Effects of forest management on haematological parameters, blood parasites, and reproductive success of the Siberian tit (Poecile cinctus) in northern Finland. Annales Zoologici Fennici 47(5): 335-346. |
| Ficedula albicollis | Passeriformes | Kulma, K. et al. 2013. Malaria infections reinforce competitive asymmetrybetween two Ficedula flycatchers in a recent contact zone. Mol. Ecol. 22(17): 4591-4601. |
| Anas discors 2 | Anseriformes | Loven, J.S. et al. 1980. Blood parasitemia in a south Texas wintering waterfowl population. J. Wildl. Dis. 16: 25-28. |
| Carduelis tristis | Passeriformes | Lumpkin, D.C. et al. 2014. Blood parasite infection differentially relates to carotenoid-based plumage and bill color in the American goldfinch. Ecol. Evol. 4(16): 3210-3217. |
| Fulmarus glacialis | Procelariiformes | Mallory, M.L. et al. 2007. Breeding status, contaminant burden and helminth parasites of Northern Fulmars Fulmarus glacialis from the Canadian high Arctic. Ibis 149(2): 338-344. |
| Charadrius alexandrinus | Charadriiformes | Martínez-de la Puente, J. et al. 2017. Extremely low Plasmodium prevalence in wild plovers and coursers from Cape Verde and Madagascar. Malaria Journal 16: 243. |
| Charadrius marginatus | Charadriiformes | Martínez-de la Puente, J. et al. 2017. Extremely low Plasmodium prevalence in wild plovers and coursers from Cape Verde and Madagascar. Malaria Journal 16: 243. |
| Charadrius pecuarius | Charadriiformes | Martínez-de la Puente, J. et al. 2017. Extremely low Plasmodium prevalence in wild plovers and coursers from Cape Verde and Madagascar. Malaria Journal 16: 243. |
| Charadrius thoracicus | Charadriiformes | Martínez-de la Puente, J. et al. 2017. Extremely low Plasmodium prevalence in wild plovers and coursers from Cape Verde and Madagascar. Malaria Journal 16: 243. |
| Cursorius cursor | Charadriiformes | Martínez-de la Puente, J. et al. 2017. Extremely low Plasmodium prevalence in wild plovers and coursers from Cape Verde and Madagascar. Malaria Journal 16: 243. |
| Larus michahellis | Charadriiformes | Martínez-Abraín, A. et al. 2002. Prevalence of blood parasites in two western-Mediterranean local populations of the Yellow-legged Gull Larus cachinnans michahellis. Ornis Fenn. 79: 34-40. |
| Philomachus pugnax | Charadriiformes | Mendes, L. et al 2013. Hidden haemosporidian infections in Ruffs (Philomachus pugnax) staging in Northwest Europe en route from Africa to Arctic Europe. Parasitol. Res. 112(5): 2037-2043. |
| Carduelis chloris | Passeriformes | Merila, J. et al. 1995. Geographic and individual variation in haematozoan infections in the greenfinch, Carduelis chloris. Canadian Journal of Zoology 73(10):1798-1804. |
| Parus caeruleus | Passeriformes | Merila, J. & Andersson, M. 1999. Reproductive effort and success are related to haematozoan infections in blue tits. Ecoscience 6(3): 421-428. |
| Anas penelope | Anseriformes | Mohammad, M.K. 2015. The Parasitic Fauna of the Wigeon Anas penelope L. 1758 Collected in central Iraq. Int. J. Adv. Res. Biol. Sci. 3(2): 243-246. |
| Acrocephalus arundinaceus 2 | Passeriformes | Nagata, H. & Sodhi, N. 2003. Low prevalence of blood parasites in five Sylviidae species in Japan. Ornithol. Sci. 2: 73–74 |
| Acrocephalus bistrigiceps | Passeriformes | Nagata, H. & Sodhi, N. 2003. Low prevalence of blood parasites in five Sylviidae species in Japan. Ornithol. Sci. 2: 73–74 |
| Cisticola juncidis | Passeriformes | Nagata, H. & Sodhi, N. 2003. Low prevalence of blood parasites in five Sylviidae species in Japan. Ornithol. Sci. 2: 73–74 |
| Locustella pryeri | Passeriformes | Nagata, H. & Sodhi, N. 2003. Low prevalence of blood parasites in five Sylviidae species in Japan. Ornithol. Sci. 2: 73–74 |
| Acrocephalus paludicola | Passeriformes | Neto, J.M. et al. 2015. Prevalence and diversity of Plasmodium and Haemoproteus parasites in the globally-threatened Aquatic Warbler Acrocephalus paludicola. Parasitology 142(9): 1183-1189. |
| Aix sponsa | Anseriformes | O’Dell, J.P. & Robbins, L.W. 1994. Hematozoa of wood ducks (Aix sponsa) in Missouri. J. Wildl. Dis 30(1): 36-39. |
| Aix sponsa 2 | Anseriformes | O’Dell, J.P. & Robbins, L.W. 1994. Hematozoa of wood ducks (Aix sponsa) in Missouri. J. Wildl. Dis 30(1): 36-39. |
| Fringilla coelebs | Passeriformes | Pawelczyk, A. et al. 2003. Parasites of chaffinch (Fringilla coelebs) population. Part ii. Blood parasites. Wiadomości parazytologiczne 49(1):31-38. |
| Delichon urbicum | Passeriformes | Piersma, T. & van der Velde, M. 2012. Dutch House Martins Delichon urbicum gain blood parasite infections over their lifetime, but do not seem to suffer. J Ornithol. 153: 907-912. |
| Picoides borealis | Piciformes | Pung, O.J. et al. 2000. Survey and host fitness effects of red-cockaded woodpecker blood parasites and nest cavity arthropods. J Parasitol 86(3):506-510. |
| Cinclus cinclus | Passeriformes | Rojo, M.A. et al. 2013. Prevalence of haematozoan parasites in the White-throated Dipper Cinclus cinclus in southern Europe. Bird Study 60(2): 247-256. |
| Dendroica coronata | Passeriformes | Rooney, L. 2015. Natural variation in malarial infection and immune investment in a migratory songbird, and the effects of infection on flight performance. Thesis (M.Sc.), University of Western Ontario. Canada. |
| Larus audouinii | Charadriiformes | Ruiz, X. et al. 1995. Incidence of a Haemoproteus lari parasitemia in a threatened Gull: Larus audouinii. Ornis Fennica 72: 159-164. |
| Parus montanus | Passeriformes | Rytkonen, S. et al. 1996. Absence of blood parasites in Willow Tits Parus montanus in northern Finland. J. Avian Biol. 27(2): 173-174. |
| Sylvia atricapilla | Passeriformes | Santiago-Alarcón, D. et al. 2011. Prevalence, diversity, and interaction patterns of avian haemosporidians in a four-year study of blackcaps in a migratory divide. Parasitology 138(7): 824-835. |
| Melanerpes carolinus | Piciformes | Schrader, M.S. et al. 2003. Seasonal prevalence of a haematozoan parasite of red-bellied woodpeckers (Melanerpes carolinus) and its association with host condition and overwinter survival. The Auk 120(1):130-137. |
| Ploceus capensis | Passeriformes | Schultz, A. et al. 2010. Infection prevalence and absence of positive correlation between avian haemosporidian parasites, mass and body condition in the Cape Weaver Ploceus capensis. Ostrich 81(1): 69-76. |
| Ploceus capensis 2 | Passeriformes | Schultz, A. et al. 2010. Infection prevalence and absence of positive correlation between avian haemosporidian parasites, mass and body condition in the Cape Weaver Ploceus capensis. Ostrich 81(1): 69-76. |
| Carduelis flammea | Passeriformes | Seutin, G. 1994. Plumage redness in redpoll finches does not reflect hemoparasitic infection. Oikos 70(2): 280-286. |
| Forpus passerinus | Psittaciformes | Sheridan, J.A. et al. 2004. Weak association between measures of health and reproductive success in green-rumped parrotlets (Forpus passerinus) in Venezuela. The Auk 121(3): 717-725. |
| Icteria virens | Passeriformes | Soares, L. et al. 2016. Co-infections of haemosporidian and trypanosome parasites in a North American songbird. Parasitology 143(14): 1930-1938. |
| Columba livia | Columbiformes | Sol, D. et al. 2000. Geographical variation in blood parasites in feral pigeons: the role of vectors. Ecography 23: 307-314. |
| Centrocercus urophasianus | Galliformes | Stabler, R.M. et al. 1977. Hematozoa in sage grouse from Colorado. J. Wildl. Dis. 13(4): 414-417. |
| Centrocercus urophasianus 2 | Galliformes | Stabler, R.M. et al. 1977. Hematozoa in sage grouse from Colorado. J. Wildl. Dis. 13(4): 414-417. |
| Emberiza citrinella | Passeriformes | Sundberg, J. 1995. Parasites, plumage coloration and reproductive success in the yellowhammer, Emberiza ctrinella. Oikos 74(2): 331-339. |
| Accipiter striatus | Accipitriformes | Taft, S.J. et al. 1996. Hematozoa in Autumnal Migrant Raptors from the Hawk Ridge Nature Reserve, Duluth, Minnesota. Helminthol. Soc. Wash. 63(1): 141-143. |
| Accipiter cooperii | Accipitriformes | Taft, S.J. et al. 1994. Avian hematozoa of adult and nestling Cooper's Hawks (Accipiter cooperii) in Wisconsin. J. Helminthol. Soc. Wash. 61(1): 146-148. |
| Accipiter cooperii 2 | Accipitriformes | Taft, S.J. et al. 1996. Hematozoa in Autumnal Migrant Raptors from the Hawk Ridge Nature Reserve, Duluth, Minnesota. Helminthol. Soc. Wash. 63(1): 141-143. |
| Accipiter gentilis | Accipitriformes | Taft, S.J. et al. 1996. Hematozoa in Autumnal Migrant Raptors from the Hawk Ridge Nature Reserve, Duluth, Minnesota. Helminthol. Soc. Wash. 63(1): 141-143. |
| Falco naumanni | Falconiformes | Tella, J.L. et al. 1996. Absence of blood-parasitization effects on Lesser Kestrel fitness. The Auk 113(1): 253-256 |
| Troglodytes troglodytes | Passeriformes | Topp, S.M. et al. 2007. Apparent absence of blood parasites in Winter Wrens in British Columbia. J. Field Ornithol. 78(3):308 313 |
| Bucanetes githagineus | Passeriformes | Valera, F. et al. 2003. Low prevalence of haematozoa in Trumpeter finches Bucanetes githagineus from south-eastern Spain: additional support for a restricted distribution of blood parasites in arid lands. Journal of Arid Environments 55: 209-213. |
| Coccothraustes coccothraustes | Passeriformes | Valkiūnas, G. et al. 2003. High prevalence of blood parasites in hawfinch Coccothraustes coccothraustes. Journal of Natural History 37:2647-2652. |
| Motacilla flava | Passeriformes | Valkiūnas, G. & Iezhova, T.A. 2001. A Comparison of the Blood Parasites in Three Subspecies of the Yellow Wagtail Motacilla flava. Journal of Parasitology 87(4): 930-934. |
| Agelaius phoeniceus | Passeriformes | Weatherhead, P.J. & Bennett, G.F. 1991. Ecology of Red-winged Blackbird parasitism by haematozoa. Canadian Journal of Zoology 69(9): 2352-2359. |
| Molothrus ater | Passeriformes | Weatherhead, P.J. & Bennett, G.F. 1992. Ecology of parasitism of Brown-headed Cowbirds by haematozoa. Canadian Journal of Zoology 70(1): 1-7. |
| Dendroica coronata 2 | Passeriformes | Weatherhead, P.J. et al. 1991. Sexual selection and parasites in wood-warblers. The Auk 108(1): 147-152. |
| Dendroica magnolia | Passeriformes | Weatherhead, P.J. et al. 1991. Sexual selection and parasites in wood-warblers. The Auk 108(1): 147-152. |
| Dendroica striata | Passeriformes | Weatherhead, P.J. et al. 1991. Sexual selection and parasites in wood-warblers. The Auk 108(1): 147-152. |
| Mniotilta varia | Passeriformes | Weatherhead, P.J. et al. 1991. Sexual selection and parasites in wood-warblers. The Auk 108(1): 147-152. |
| Oporornis philadelphia | Passeriformes | Weatherhead, P.J. et al. 1991. Sexual selection and parasites in wood-warblers. The Auk 108(1): 147-152. |
| Seiurus aurocapilla | Passeriformes | Weatherhead, P.J. et al. 1991. Sexual selection and parasites in wood-warblers. The Auk 108(1): 147-152. |
| Seiurus noveboracensis | Passeriformes | Weatherhead, P.J. et al. 1991. Sexual selection and parasites in wood-warblers. The Auk 108(1): 147-152. |
| Setophaga ruticilla | Passeriformes | Weatherhead, P.J. et al. 1991. Sexual selection and parasites in wood-warblers. The Auk 108(1): 147-152. |
| Vermivora peregrina | Passeriformes | Weatherhead, P.J. et al. 1991. Sexual selection and parasites in wood-warblers. The Auk 108(1): 147-152. |
| Wilsonia pusilla | Passeriformes | Weatherhead, P.J. et al. 1991. Sexual selection and parasites in wood-warblers. The Auk 108(1): 147-152. |
| Parus atricapillus | Passeriformes | Wilkinson, L.C. et al. 2016. Avian malaria in a boreal resident species: long-term temporal variability, and increased prevalence in birds with avian keratin disorder. Int. J. Parasitol. 46(6): 281-290. |
| Fregata minor | Suliformes | Work, T.M. & Rameyer, R.A. 1996. Haemoproteus iwa n. sp. in Great Frigatebirds (Fregata minor [Gmelin]) from Hawaii: Parasite Morphology and Prevalence. J. Parasitol. 82(3): 489-491. |
| Calidris melanotos | Charadriiformes | Yohannes, E. et al. 2009. Prevalence of malaria and related haemosporidian parasites in two shorebird species with different winter habitat distribution. J. Ornithol. 150: 287-291. |
| Calidris pusilla | Charadriiformes | Yohannes, E. et al. 2009. Prevalence of malaria and related haemosporidian parasites in two shorebird species with different winter habitat distribution. J. Ornithol. 150: 287-291. |
| Larus argentatus | Charadriiformes | Zagalska-Neubauer, M. et al. 2016. High prevalence of Leucocytozoon parasites in fresh water breeding gulls. J. Ornithol. 157: 525-532. |

**Table S2**. List of 39 studies consulted to extract data on gastrointestinal parasites.

| species | order | study |
| --- | --- | --- |
| Galerida_cristata | Passeriformes | Al-Ankari, A.-R.S. et al. 2003. First Report of Variolepis farciminosa (Cestoda: Hymenolepididae) and Diplotriaena tridens (Nematoda: Diplotriaenoidea) Infecting Crested Larks, Galerida cristata, from Hofuf, Al-Ahsa Oasis, Saudi Arabia. Comp. Parasitol. 70(1): 97-98. |
| Larus_michahellis | Charadriiformes | Álvarez, M.F. et al. 2006. Influence of host age and sex on the helminth fauna of the yellow-legged gull Larus Michahellis in Galicia Northwestern Spain. J Paratisol. 92(3): 454-458. |
| Pica_pica | Passeriformes | Amin, O.M. et al. 2010. Redescription of Sphaerirostris picae (Acanthocephala: Centrorhynchidae) from magpie, Pica pica, in Northern Iran, with special reference to unusual receptacle structures and notes on histopathology. 96(3): 561-568. |
| Anser_albifrons | Anseriformes | Amundson, C.L. et al. 2016. Helminth community structure in two species of arctic-breeding waterfowl. Int. J. Parasitol. Parasites Wildl. 5(3): 263-272. |
| Branta_bernicla | Anseriformes | Amundson, C.L. et al. 2016. Helminth community structure in two species of arctic-breeding waterfowl. Int. J. Parasitol. Parasites Wildl. 5(3): 263-272. |
| Haematopus_ostralegus | Charadriiformes | Borgsteede, F.H.M. et al. 1988. Helminth parasites of the digestive tract of the oystercatcher, Haematopus ostralegus, in the Wadden Sea, The Netherlands. Neth. J. Sea Res. 22(2): 171-174. |
| Uria_aalge | Charadriiformes | Brosens, L. et al. 1996. Observations on the helminths of harbour porpoises (Phocoena phocoena) and common guillemots (Uria aalge) from the Belgian and German coasts. Vet. Record 139(11): 254-257. |
| Carduelis_chloris | Passeriformes | Brown, M.A. et al. 2010. Coccidian parasites of British wild birds. J. Nat. Hist. 44(43-44): 2669-2691. |
| Emberiza_citrinella | Passeriformes | Brown, M.A. et al. 2010. Coccidian parasites of British wild birds. J. Nat. Hist. 44(43-44): 2669-2691.. |
| Fringilla_coelebs | Passeriformes | Brown, M.A. et al. 2010. Coccidian parasites of British wild birds. J. Nat. Hist. 44(43-44): 2669-2691. |
| Parus_caeruleus | Passeriformes | Brown, M.A. et al. 2010. Coccidian parasites of British wild birds. J. Nat. Hist. 44(43-44): 2669-2691. |
| Parus_major | Passeriformes | Brown, M.A. et al. 2010. Coccidian parasites of British wild birds. J. Nat. Hist. 44(43-44): 2669-2691. |
| Passer_domesticus | Passeriformes | Brown, M.A. et al. 2010. Coccidian parasites of British wild birds. J. Nat. Hist. 44(43-44): 2669-2691. |
| Phylloscopus_trochilus | Passeriformes | Brown, M.A. et al. 2010. Coccidian parasites of British wild birds. J. Nat. Hist. 44(43-44): 2669-2691. |
| Prunella_modularis | Passeriformes | Brown, M.A. et al. 2010. Coccidian parasites of British wild birds. J. Nat. Hist. 44(43-44): 2669-2691. |
| Regulus_regulus | Passeriformes | Brown, M.A. et al. 2010. Coccidian parasites of British wild birds. J. Nat. Hist. 44(43-44): 2669-2691. |
| Sturnus_vulgaris | Passeriformes | Brown, M.A. et al. 2010. Coccidian parasites of British wild birds. J. Nat. Hist. 44(43-44): 2669-2691. |
| Sylvia_atricapilla | Passeriformes | Brown, M.A. et al. 2010. Coccidian parasites of British wild birds. J. Nat. Hist. 44(43-44): 2669-2691. |
| Anas_acuta | Anseriformes | Crichton, V.F.J. & Welch, H.E. 1972. Helminths from the digestive tracts of mallards and pintails in the Delta Marsh, Manitoba. Canadian Journal of Zoology 50(5): 633-637. |
| Anas_platyrhynchos | Anseriformes | Crichton, V.F.J. & Welch, H.E. 1972. Helminths from the digestive tracts of mallards and pintails in the Delta Marsh, Manitoba. Canadian Journal of Zoology 50(5): 633-637. |
| Callipepla_squamata | Galliformes | Dunham, N.R. & Kendall, R.J. 2016. Eyeworm infections of Oxyspirura petrowi, Skrjabin, 1929 (Spirurida: Thelaziidae), in species of quail from Texas, New Mexico and Arizona, USA. J. Helminthol. 91(4): 491-496. |
| Colinus_virginianus | Galliformes | Dunham, N.R. et al. 2014. Evidence of an Oxyspirura petrowi epizootic in northern bobwhites (Colinus virginianus), Texas, USA. J. Wildl. Dis. 50(3): 552-558. |
| Quiscalus_quiscula | Passeriformes | Fayer, R. & Kocan, R.M. 1971. Prevalence of Sarcocystis in Grackles in Maryland. Journal of Protozoology 18(3): 547-548. |
| Chen_caerulescens | Anseriformes | Gajadhar, A. et al. 1983. Prevalence of renal coccidia in wild waterfowl in Saskatchewan. Canadian Journal of Zoology 61(11): 2631-2633. |
| Dendrocygna_autumnalis | Anseriformes | George, R.R. & Bolen, E.G. 1975. Endoparasites of black-bellied whistling ducks in Southern Texas. J. Wildl. Dis. 11(1): 17-22. |
| Phasianus_colchicus | Galliformes | Gethings, O.J. et al. 2016. Body condition is negatively associated with infection with Syngamus trachea in the ring-necked pheasant (Phasianus colchicus). Vet. Parasitol. 228: 1-5. |
| Anas_crecca | Anseriformes | Green, A.J. et al. 2011. Determinants of the prevalence of the cloacal cestode Cloacotaenia megalops in teal wintering in the French Camargue. Eur. J. Wildl. Res. 57: 275–281. |
| Larus_argentatus | Charadriiformes | Gysels, H. & Rabaey, M. 1964. The incidence of some species of trematoda in three species of Larus gulls in Wales. Ibis 106(4): 532-540. |
| Larus_argentatus_2 | Charadriiformes | Gysels, H. & Rabaey, M. 1964. The incidence of some species of trematoda in three species of Larus gulls in Wales. Ibis 106(4): 532-540. |
| Larus_fuscus | Charadriiformes | Gysels, H. & Rabaey, M. 1964. The incidence of some species of trematoda in three species of Larus gulls in Wales. Ibis 106(4): 532-540. |
| Larus_fuscus_2 | Charadriiformes | Gysels, H. & Rabaey, M. 1964. The incidence of some species of trematoda in three species of Larus gulls in Wales. Ibis 106(4): 532-540. |
| Larus_marinus | Charadriiformes | Gysels, H. & Rabaey, M. 1964. The incidence of some species of trematoda in three species of Larus gulls in Wales. Ibis 106(4): 532-540. |
| Larus_marinus_2 | Charadriiformes | Gysels, H. & Rabaey, M. 1964. The incidence of some species of trematoda in three species of Larus gulls in Wales. Ibis 106(4): 532-540. |
| Tympanuchus_cupido | Galliformes | Harper, G.R. et al. 1967. Helminths of greater prairie chickens in Kansas. The Journal of Wildlife Management 31(2): 265-269. |
| Lagopus_lagopus | Galliformes | Holmastad, P.R. & Skorping A. 1998. Covariation of parasite intensities in willow ptarmigan, Lagopus lagopus L. Canadian Journal of Zoology 76(8): 1581-1588. |
| Tetrao_tetrix | Galliformes | Isomursu, M. et al. 2006. Sex and age influence intestinal parasite burden in three boreal grouse species. Journal of Avian Biology, 37: 516-522 |
| Tetrao_urogallus | Galliformes | Isomursu, M. et al. 2006. Sex and age influence intestinal parasite burden in three boreal grouse species. Journal of Avian Biology, 37: 516-522 |
| Phalacrocorax_carbo | Suliformes | Kanarek, G. & Zalesny, G. 2013. Extrinsic- and intrinsic-dependent variation in component communities and patterns of aggregations in helminth parasites of great cormorant (Phalacrocorax carbo) from N.E. Poland. Parasitol. Res. 113: 837-850. |
| Larus_delawarensis | Charadriiformes | Levy, M.S. 1997. Helminth communities of ring-billed gull (Larus delawerensis) collected along the St. Lawrence River and Estuary. Thesis (M.Sc.), Concordia University. Canada. |
| Tyrannus_tyrannus | Passeriformes | Mackenzie, D.I. et al. 1979. Comparison of the helminth fauna of eastern and western kingbirds at Delta Marsh, Manitoba. Canadian Journal of Zoology 57(5): 1143-1149. |
| Tyrannus_verticalis | Passeriformes | Mackenzie, D.I. et al. 1979. Comparison of the helminth fauna of eastern and western kingbirds at Delta Marsh, Manitoba. Canadian Journal of Zoology 57(5): 1143-1149. |
| Fulmarus_glacialis | Procellariiformes | Mallory, M.L. et al. 2007. Breeding status, contaminant burden and helminth parasites of Northern Fulmars Fulmarus glacialis from the Canadian high Arctic. Ibis 149(2): 338-344. |
| Fulmarus_glacialis_2 | Procellariiformes | Mallory, M.L. et al. 2007. Breeding status, contaminant burden and helminth parasites of Northern Fulmars Fulmarus glacialis from the Canadian high Arctic. Ibis 149(2): 338-344. |
| Strix_aluco | Strigiformes | McInes, F.J. et al. 1994. The distribution of Centrorhynchus aluconis (Acanthocephala) and Porrocaecum spirale (Nematoda) in tawny owls (Strix aluco) from Great Britain. J. Raptor Res. 28(1): 34-38. |
| Anas_platyrhynchos_2 | Anseriformes | Mercado-Reyes, M. et al. 2010. Presence of helminths in the Mexican duck Anas platyrhynchos diazi of the Zacatecano Plateau, Mexico. Agrociencia 44(8): 931-939. |
| Corvus_frugilegus | Passeriformes | Mettrick, D.F. 1960 Helminth Parasites of Hertfordshire Birds—IV Survey Results. Journal of Helminthology 43(3-4): 267-276. |
| Corvus_monedula | Passeriformes | Mettrick, D.F. 1960 Helminth Parasites of Hertfordshire Birds—IV Survey Results. Journal of Helminthology 43(3-4): 267-276. |
| Alectoris_rufa | Galliformes | Millán, J. et al. 2004. Ecology of nematode parasitism in red-legged partridges (Alectoris rufa) in Spain. Helminthologia 41(1): 33-37. |
| Anas_penelope | Anseriformes | Mohammad, M.K. 2015. The Parasitic Fauna of the Wigeon Anas penelope L. 1758 Collected in central Iraq. Int. J. Adv. Res. Biol. Sci. 3(2): 243-246. |
| Sturnus_vulgaris_2 | Passeriformes | Moore, J. & Bell, D.H. 1983. Pathology (?) of Plagiorhynchus cylindraceus in the starling, Sturnus vulgaris. J. Parasitol. 69(2): 387-390. |
| Callipepla_californica | Galliformes | Moore, J. et al. 1988. Dispharynx nasuta (Nematoda) in California Quail (Callipepla californica) in Western Oregon. J. Wildl. Dis. 24(3): 564-567. |
| Branta_canadensis | Anseriformes | Nowicki, A. et al. 1995. Gizzard nematodes of Canada geese wintering in Southern Illinois. J. Wildl. Dis. 31(3): 307-313. |
| Sialia_sialis | Passeriformes | Carleton, R.E. 2007. Disease ecology of a population of eastern bluebirds (Sialia sialis) in Georgia: a starting point. Thesis (PhD), University of Georgia. USA. |
| Sula_nebouxii | Suliformes | Rubio-Godoy, M. et al. 2011. Helminth Parasites of the Blue-Footed Booby on Isla Isabel, México. J. Parasitol. 97(4): 636-641. |
| Aythya_fuligula | Anseriformes | Rzad, I. et al. 2012. Digenean communities in the tufted duck [Aythya fuligula (L., 1758)] and greater scaup [A. marila (L., 1761)] wintering in the north-west of Poland. J. Helmonthol. 87(2): 230-239. |
| Aythya_marila | Anseriformes | Rzad, I. et al. 2012. Digenean communities in the tufted duck [Aythya fuligula (L., 1758)] and greater scaup [A. marila (L., 1761)] wintering in the north-west of Poland. J. Helmonthol. 87(2): 230-239. |
| Scolopax_rusticola | Charadriiformes | Sanchez-Garcia, C. et al. 2017. Is cestode infection intensity associated with decreased body condition in the Eurasian woodcock Scolopax rusticola? J. Helminthol. 92(1): 42-48. |
| Vanellus_chilensis | Charadriiformes | Silveira, T.S. & Calegaro-Marques, C. 2016. Helminth parasite diversity discloses age and sex differences in the foraging behaviour of southern lapwings Vanellus chilensis. Austral Ecology 41(5): 549-558. |
| Ciconia_ciconia | Ciconiiformes | Sitko, J. & Heneberg, P. 2015. Composition, structure and pattern of helminth assemblages associated with central European storks (Ciconiidae). Parasitology International 64(2): 130-134. |
| Anas_crecca_2 | Anseriformes | Turner, B.C. & W. Threlfall. 1975. The Metazoan parasites of Green-winged Teal (Anas crecca L.) and Blue-winged Teal (Anas discors L.) from eastern Canada. Proc. Helminthol. Soc. Wash .42: 157-169. |
| Columba_livia | Columbiformes | Vaz, FF. et al. 2017. Gastrointestinal helminths of two populations of wild pigeons Columba livia in Brazil. Rev. Bras. Parasitol. Vet. 26(4): 446-450. |
